# The missing piece in inclusion: addressing school avoidance among children with autism

**DOI:** 10.3389/fpsyg.2026.1724420

**Published:** 2026-02-17

**Authors:** Ane Dorthe Berg, Kristine Sørløk, Ann-Kathrin Bretfeld-Wolf, Alexander Gamst Page, Marianne Hatlem Storås, Sobh Chahboun

**Affiliations:** 1 Faculty of Social and Educational Sciences, Department of Education and Lifelong Learning, Norwegian University of Science and Technology, Trondheim, Norway; 2 Åsveien School and Resource Center, Trondheim, Norway; 3 Child and Adolescent Psychiatric Outpatient Clinic, Trondheim, Norway; 4 Queen Maud University College, Trondheim, Norway; 5 Faculty of Social and Educational Sciences, Department of Social Work (ISA), Norwegian University of Science and Technology, Trondheim, Norway; 6 Faculty of Education and International Studies, Department of Early Childhood Education, Oslo Metropolitan University, Oslo, Norway

**Keywords:** Autism Spectrum Disorder (ASD), school avoidance, inclusive education, emotional support, individualized interventions

## Abstract

This current article examines the relationship between Autism Spectrum Disorder (ASD) and school avoidance, focusing on the theoretical and practical challenges involved. ASD, a neurodevelopmental condition, presents significant challenges in social interaction, communication, and restricted and repetitive behaviors, which can hinder educational engagement. School avoidance, characterized by elevated absenteeism and emotional distress, emerges as a critical issue for many students with ASD. By exploring historical foundations, diagnostic classification systems, and the core characteristics and challenges of ASD, this analysis highlights factors that contribute to school avoidance. The distinction between school avoidance and truancy is emphasized, underscoring the importance of tailored interventions that address sensory sensitivities, emotional wellbeing, and the need for structured and predictable environments. Practical strategies for inclusion, teacher–student relationships, school–home collaboration, and interdisciplinary cooperation are also discussed, offering a framework for creating supportive educational settings. Ultimately, this work connects theoretical insights with actionable practices to promote engagement, reduce stress, and support the development of students with ASD.

## Introduction

Autism Spectrum Disorder (ASD) is a neurodevelopmental condition that significantly impacts an individual's school functioning, affecting social interaction, communication, and behavioral patterns (Helverschou, 2022). Research has consistently shown that individuals with ASD are at a heightened risk of school avoidance behaviors, often manifested as reluctance to attend school, frequent absenteeism, or refusal to engage in school-related activities (Havik, 2018; Martnes, 2022; Munkhaugen, 2019). Such avoidance can be attributed to a variety of factors, including sensory sensitivities, difficulties in understanding social cues, and challenges in adapting to changes in routine or environmental stressors (Nordin et al., 2024; Chahboun et al., 2024).

The connection between autism and school avoidance can be understood through a theoretical lens that considers both the individual characteristics of children with ASD and the external demands of the school environment. For example, the cognitive rigidity and preference for sameness often observed in individuals with autism may lead to difficulties in managing unexpected changes in daily routines, which can result in increased stress and avoidance behaviors. Additionally, social communication challenges—such as difficulties in forming peer relationships and interpreting social cues—may lead to feelings of isolation, further contributing to reluctance to attend school (Helverschou, 2022).

To address these challenges, it is crucial to implement targeted interventions and accommodations tailored to the unique needs of students with ASD. This includes providing structured routines, clear expectations, and visual supports to reduce anxiety and enhance predictability in the school day (Statped, 2021, 2022). Social skills training and the creation of a supportive school climate—where students feel safe, included, and understood—are also essential components in helping children with ASD overcome school avoidance behaviors.

This article explores the complex interplay between autism and school avoidance, drawing on historical, diagnostic, and practical perspectives. By examining the foundational theories of autism, the core characteristics associated with ASD, and the specific factors contributing to school avoidance, this analysis seeks to provide a comprehensive framework for addressing these challenges. It emphasizes the importance of inclusive practices, tailored interventions, and collaborative efforts to create educational environments that promote engagement and wellbeing for students with ASD.

### Autism: historical and theoretical foundations

Leo Kanner's seminal work in 1943 laid the foundation for understanding autism as a distinct condition. He identified key characteristics of what he termed “early infantile autism,” highlighting the presence of symptoms from early childhood (Øzerk and Øzerk, 2013; Helverschou, 2022). Around the same time, Hans Asperger described a similar developmental profile, emphasizing the spectrum nature of autism and its complex interplay of biological and environmental factors (Øzerk and Øzerk, 2013).

The contrasting perspectives of Asperger and Bettelheim—the latter attributing autism to maternal neglect—highlighted early debates in autism research. Lorna Wing's introduction of the term “autism spectrum” in 1981 expanded the understanding of autism to include a continuum of developmental presentations. Her work, alongside Michael Rutter's research from 1970 validating autism as a diagnostic category, emphasized the heterogeneity of the condition. These foundational theories have informed contemporary diagnostic practices, which recognize autism as encompassing individuals with a wide range of cognitive and communicative abilities (Helverschou, 2022).

These early theoretical contributions also laid the groundwork for recognizing the need for multifaceted intervention approaches. The shift from perceiving autism as a static condition to understanding it as a dynamic spectrum has been crucial in tailoring educational and therapeutic practices to individual needs and in acknowledging that support requirements may change across developmental stages and educational transitions.

## Defining autism spectrum disorder

ASD is currently defined using diagnostic classification systems such as the *Diagnostic and Statistical Manual of Mental Disorders, Fifth Edition* (DSM-5; American Psychiatric Association, 2013) and the *International Classification of Diseases, 11th Revision* (ICD-11; World Health Organization, 2019). These diagnostic systems characterize autism by persistent challenges in social communication and interaction, alongside restricted, repetitive patterns of behavior, interests, or activities (Chahboun et al., 2022).

The DSM-5 and ICD-11 emphasize the significant variability in how ASD manifests, influenced by factors such as co-occurring conditions, cognitive abilities, and sensory processing differences. Both diagnostic systems highlight the importance of considering comorbidities—including anxiety, depression, and attention deficit hyperactivity disorder (ADHD)—in understanding the broader impact of autism on daily functioning. In addition, the DSM-5 and ICD-11 underscore the necessity of assessing atypical sensory responses, which often contribute to heightened stress levels in individuals with ASD (Fredriksen et al., 2025).

A critical implication of these diagnostic systems is the acknowledgment of the dynamic nature of ASD-related characteristics over time. This reinforces the need for continuous monitoring and adaptation of support strategies to align with developmental changes and contextual challenges faced by individuals with autism, including transitions within and between educational settings.

According to Oslo universitetssykehus HF (2025), people with autism can experience both physical and mental health challenges, and they may also have other neurodevelopmental conditions such as intellectual disability and attention deficit hyperactivity disorder (ADHD) (Chahboun et al., 2024). There is considerable variation in the severity of these additional challenges, although the highest rates are seen among those with intellectual disabilities (Helverschou, 2022; Bakken, 2022). Somatic complaints such as stomach pain, sleep difficulties, and other gastrointestinal problems are relatively common, as is epilepsy, particularly among individuals with both autism and intellectual disability. Mental health conditions, especially anxiety and depression, are also prevalent (Bakken, 2022; Martnes, 2022).

Many of these difficulties—such as anxiety, sleep problems, and somatic complaints—are not unique to students with autism; they are also observed among neurotypical students experiencing stress and anxiety. However, in autistic students they may occur more frequently and interact with sensory sensitivities and social challenges, thereby increasing vulnerability to school avoidance (Havik, 2018; Martnes, 2022). These co-occurring conditions may sometimes be due to shared biological factors, while in other cases they may arise as secondary effects of growing up with autism in environments that do not sufficiently accommodate neurodiversity. Accurate assessment therefore requires expertise in both autism and mental health, as well as careful differential diagnosis when identifying co-occurring disorders (Fredriksen et al., 2025).

## Core features and challenges in autism

### Social interaction

Differences and challenges in social interaction are a fundamental characteristic of ASD and are evident in several key areas, including interpreting social cues, maintaining eye contact, and engaging in reciprocal social interactions. These social-communication differences are typically noticeable by the age of two and can have a profound impact on an individual's ability to form and sustain meaningful relationships (Helverschou, 2022).

For children with ASD, difficulties in recognizing or responding appropriately to emotions—their own and those of others—make it hard to navigate social situations. As a result, they may experience frequent misunderstandings, which can contribute to a sense of social isolation and alienation from peers. Social interaction challenges often intersect with other core features, exacerbating the struggles faced by individuals with ASD. The inability to connect socially is not only distressing for the child; it can also interfere with developmental progress in areas such as communication, self-regulation, and learning.

Social skills are integral to various aspects of life, including forming friendships, maintaining family relationships, participating in group activities, and engaging in academic settings. Misinterpretations of social cues can hinder the development of peer relationships, creating a cycle of isolation that may impact mental health and educational engagement. For example, a child who struggles to understand jokes, sarcasm, or implicit social rules may be perceived as “odd” or withdrawn, increasing the risk of bullying and exclusion (Fredriksen et al., 2025).

Moreover, social interaction challenges frequently intersect with communication and sensory processing differences. An individual who is hypersensitive to sensory input may become overwhelmed in busy social settings, making it harder to focus on or respond to the social context. Difficulties in reading facial expressions, gestures, and tone of voice can further complicate these interactions, increasing stress and contributing to school avoidance when social demands in the school environment feel unmanageable (Fredriksen et al., 2025).

### Communication challenges

Language and communication in individuals with ASD are highly heterogeneous, reflecting a broad spectrum of abilities and needs. Some individuals may have severely limited spoken language or be entirely non-verbal, relying on augmentative and alternative communication (AAC) methods—such as picture exchange systems, sign language, or communication devices—to express themselves. Others may demonstrate advanced vocabulary, fluent speech, and complex sentence structures, yet still experience significant challenges with the pragmatic (social) aspects of language (Øzerk and Øzerk, 2013; Skogli-Christensen et al., 2025).

Pragmatic language differences often manifest as difficulties in understanding and using language flexibly in social situations. Individuals with ASD may struggle with non-literal language—such as metaphors, idioms, sarcasm, and indirect requests—which rely heavily on shared cultural and contextual knowledge (Skogli-Christensen et al., 2025). This can lead to literal interpretations of figurative expressions and misunderstandings in everyday interactions. These challenges are often compounded by difficulties in recognizing and interpreting non-verbal cues, such as facial expressions, gestures, body language, and prosody (Smart et al., 2025).

Individuals with ASD may also experience difficulties in initiating, maintaining, and appropriately concluding conversations. Challenges can include turn-taking, staying on topic, or adjusting communication style to suit different social contexts or conversation partners. Such difficulties may lead to social frustration and anxiety, contributing to withdrawal or avoidance in social and academic settings, including classroom discussions and group work (Balik and Ozgun, 2024). Addressing these multifaceted communication challenges requires individualized, context-sensitive interventions tailored to the strengths and needs of each person (Skogli-Christensen et al., 2025). Effective strategies may include structured social skills training, explicit instruction in pragmatic language use, and the incorporation of visual supports to enhance comprehension. Interventions should also emphasize the development of social-emotional understanding and the ability to interpret non-verbal communication cues. A multidisciplinary approach—engaging educators, speech-language pathologists, psychologists, and family members—is often essential in creating supportive environments that foster both linguistic competence and social connectedness for individuals with ASD (Skogli-Christensen et al., 2025).

### Repetitive behaviors and restricted interests

Individuals with ASD often display a range of repetitive behaviors and highly focused interests, which are core diagnostic features of the condition (Helverschou, 2022). These behaviors may include repetitive motor movements (e.g., hand-flapping, rocking), repetitive use of objects, insistence on sameness, adherence to routines, and intense, circumscribed interests that may appear unusual in focus or intensity.

Rigid adherence to routines and pronounced resistance to change can present significant challenges in dynamic environments such as schools, where flexibility and adaptability are often required. However, it is important to recognize that these repetitive behaviors and restricted interests (RRBs) are not merely “problem behaviors” but often serve important self-regulatory functions. RRBs can act as coping mechanisms that help individuals manage heightened stress, anxiety, and sensory overload in overstimulating environments, including classrooms and schoolyards. Engaging in known routines or interests may provide predictability and a sense of control in a world that can otherwise feel chaotic.

Research suggests that anxiety can be a catalyst for RRBs and may influence their frequency and rigidity; during periods of overwhelm, RRBs may become more evident or inflexible as a way of maintaining emotional equilibrium and predictability (e.g., Jiujias et al., 2017; Joyce et al., 2017; Richler et al., 2010). In this sense, RRBs may provide a sense of wellbeing and consistency for students with ASD when they face situations that heighten anxiety (Attwood, 2007; Spiker et al., 2012). For educators, adopting a nuanced understanding of the function of RRBs is crucial. Rather than viewing them solely as behaviors to be eliminated, it may be more helpful to reduce environmental stressors and support alternative, less disruptive strategies for self-regulation (Fredriksen et al., 2025).

Educational interventions that thoughtfully balance the need for structure with opportunities for flexibility can significantly reduce distress and promote wellbeing for students with ASD. This approach not only minimizes behavioral disruptions but also fosters an inclusive learning environment where neurodiverse students' coping strategies are understood and accommodated.

## School avoidance in children with autism

School avoidance is an increasingly prevalent concern among children with ASD (Havik, 2018; Martnes, 2022; Munkhaugen, 2019). It is characterized by persistent difficulties in attending or remaining in school, often resulting in chronic absenteeism and significant distress, which can have long-term consequences for academic achievement, social development, and mental health.

Children with ASD frequently experience heightened emotional distress in response to the social, sensory, and academic demands of school. Sensory sensitivities—such as hypersensitivity to noise, bright lights, crowded spaces, or certain textures—can create overwhelming experiences that contribute to anxiety and avoidance. Inadequate support systems and a lack of individualized accommodations exacerbate these challenges, making the school environment feel unpredictable and threatening rather than safe and supportive.

Havik (2018) identifies somatic and psychosomatic symptoms commonly observed in children who exhibit school avoidance, including recurrent nausea, stomach pain, headaches, and general malaise, particularly in the mornings before school or evenings before the next school day (Pavlopoulou et al., 2025). These physical symptoms often diminish or disappear when the child is not required to attend school, suggesting a strong link between somatic complaints and psychological distress associated with the school environment.

School avoidance among children with ASD is frequently associated with various forms of anxiety, including social anxiety, separation anxiety, performance anxiety, and exposure anxiety (Havik, 2018; Martnes, 2022). Depression is also commonly co-occurring, further complicating the child's ability to engage with school. In some cases, school avoidance may occur even in the absence of clinically significant anxiety, for example when students experience persistent social difficulties, peer rejection, bullying, or a lack of belonging.

Addressing school avoidance in children with ASD requires a comprehensive, individualized approach that considers emotional, sensory, and social factors. Early identification and intervention are critical to prevent escalation. Strategies may include creating more predictable and supportive school environments, implementing sensory-friendly accommodations, providing access to mental health support, and fostering strong school–home collaboration. Gradual reintroduction to school, with flexible schedules and carefully scaffolded expectations, can help reduce anxiety and build coping skills, promoting long-term engagement and wellbeing.

### Distinguishing school avoidance from truancy

School avoidance differs fundamentally from truancy, both in underlying causes and in implications for intervention. Truancy is typically characterized by deliberate absenteeism associated with low interest in academic activities, defiance of authority, or engagement in alternative activities outside school. In contrast, students experiencing school avoidance often *want* to attend school but are hindered by significant emotional distress, sensory processing difficulties, or other psychological barriers (Havik, 2018).

These students may experience intense anxiety related to specific aspects of the school environment, such as social interactions, academic pressures, or sensory overstimulation. Forcing attendance without addressing these underlying factors may intensify distress and strengthen avoidance (Lukito et al., 2025). Conflating school avoidance with truancy can therefore lead to inappropriate interventions, such as punitive measures and behavior-focused attendance monitoring, which are largely ineffective—and potentially harmful—for students with school avoidance, particularly those with ASD (Lukito et al., 2025).

Effective responses to school avoidance must prioritize emotional wellbeing and address the specific factors that contribute to distress. Interventions may include cognitive-behavioral therapy (CBT), anxiety management strategies, structured and predictable classroom routines, and adjustments to reduce sensory overload. Collaboration among educators, school-based support services, mental health professionals, and families is essential to ensure that interventions are comprehensive, consistent, and sensitive to the child's needs.

Rather than relying on external accountability alone, strategies for school avoidance should focus on building internal coping mechanisms, resilience, and a sense of belonging within the school community. By addressing both emotional and environmental dimensions of school avoidance, interventions can promote sustained engagement and support students' development and wellbeing.

## Challenges for children with autism and school avoidance

Children with ASD often face significant challenges related to executive functioning, which encompasses cognitive processes essential for goal-directed behavior. These challenges may involve problem-solving, planning, organizing, task initiation and completion, and emotional regulation (Nordby, 2019). Difficulties in these areas can affect a child's ability to manage complex tasks, adapt to new situations, and respond flexibly to changes in routines.

In addition, many children with ASD experience communication and social understanding challenges—such as interpreting abstract language (idioms, sarcasm), understanding unwritten social rules, recognizing others' perspectives (theory of mind), and accurately reading intentions or emotions in others' behavior. These combined challenges often result in uncertainty about what is expected in different academic and social contexts.

When students lack clear structure and predictability in their daily routines, stress and anxiety may increase considerably. Many students with ASD already enter the schoolyard with elevated levels of stress or anxiety, and these levels can fluctuate depending on the support they receive and on daily events such as examinations, specific subjects, teacher changes, weather (e.g., storms), and social pressures (Havik, 2018; Statped, 2022). The absence of a consistent and supportive environment may leave children feeling overwhelmed and unsure of how to cope.

This stress can manifest in a variety of behavioral responses, including cognitive rigidity, compulsive routines, oppositional behaviors, and pronounced anxiety. These behaviors are often attempts to regain a sense of control rather than deliberate defiance. For some children with ASD, controlling behaviors—such as insisting on rigid routines, perfectionism, or avoiding stressful environments—serve as self-regulation strategies to manage anxiety and sensory overload. Over time, however, such strategies may contribute to social withdrawal and exclusion, as interactions with peers and teachers become increasingly strained (Nordby, 2019).

For some children, the cumulative effect of these stressors leads to school avoidance as a coping mechanism. Staying at home provides immediate relief and a sense of safety, even though it does not address underlying difficulties. Havik (2018) emphasizes that for many children with ASD, avoiding school is not rooted in defiance or disinterest, but in attempts to protect themselves from overwhelming stressors they cannot effectively manage within the school setting.

Supporting students with ASD therefore requires individualized, student-centered interventions that address executive functioning, communication, and sensory needs. Early identification of challenges and timely, tailored accommodations are critical to preventing the escalation of difficulties, including the development of entrenched school avoidance patterns. Thorough, multidisciplinary assessments—combined with close collaboration with parents—are essential for identifying specific needs and designing interventions that are both relevant and effective (Ashworth et al., 2025).

Interventions should focus on identifying triggers for distress and implementing strategies to mitigate them. Such strategies may include structured routines, visual schedules, clear expectations, sensory accommodations, and targeted social skills training. Providing a predictable and supportive learning environment can significantly reduce anxiety, promote engagement, and foster a sense of competence and belonging for students with ASD (Statped, 2021, 2022). Without proactive and individualized support, the risk of chronic school avoidance and long-term academic and social difficulties increases substantially (Nordby, 2019).

### Consequences of school avoidance for children with autism

School avoidance is a complex and multifaceted phenomenon that poses significant stress for children, their families, and schools. It has both immediate and long-term consequences for social, emotional, and educational development, affecting not only academic progress but also personal growth and wellbeing (Havik, 2018). The longer a child remains absent from school, the more difficult it becomes to facilitate reintegration, partly due to the reinforcing cycle of avoidance: the temporary relief experienced by staying home strengthens the avoidance behavior and increases psychological barriers to returning (Fremont, 2003).

Prolonged absenteeism can lead to learning gaps, reduced academic achievement, and diminished self-efficacy, which in turn lowers motivation to engage with school-related tasks. Emotionally, chronic absenteeism may contribute to low self-worth, increased stress, and anxiety—especially when students feel they are falling behind or are disconnected from peers. Socially, extended absence from the structured environment of school can lead to isolation, weakened peer relationships, and difficulties in developing and maintaining key social skills (Havik, 2018).

In the long term, the consequences extend beyond schooling. Individuals with a history of persistent absenteeism are at increased risk of financial challenges, difficulties in obtaining and maintaining employment, and enduring mental health problems such as anxiety and depression. The loss of structured daily routines during formative years can hinder the development of essential life skills, complicating the transition to independent adult roles.

These potential outcomes underscore the importance of early and effective intervention to stabilize school attendance. Even sporadic absences can set a precedent that normalizes avoidance. Preventative strategies should focus on identifying at-risk students early, understanding the maintaining factors that perpetuate school avoidance, and implementing tailored support plans that address cognitive, emotional, social, and environmental contributors (Nordby, 2019). Multidisciplinary collaboration among educators, mental health professionals, parents (Ashworth et al., 2025), and students is often necessary to create individualized plans that support regular attendance, promote emotional wellbeing, and foster a positive connection to school.

## The role of stress and sensory sensitivities

Children with ASD often experience heightened stress due to unpredictable or overwhelming environments. Disruptions in routines and sensory overload can lead to behavioral outbursts and longer-term emotional challenges (Statped, 2022). Accommodations that address sensory triggers—such as reducing noise, providing quiet spaces, or using visual schedules—can significantly reduce stress and promote engagement.

Elvèn (2009) describes fundamental stress factors that frequently contribute to high stress levels among individuals with ASD. These factors are present in everyday life and may include sleep problems, lack of structure and predictability, difficulties in social understanding, and sensory overload (related to light, sound, smell, taste, and touch). Above these fundamental stressors are situational stressors, many of which involve disruptions to routines. Even seemingly minor changes, such as wearing different clothes than expected or unexpected changes in timetable, can be challenging for some individuals on the autism spectrum.

These stressors can significantly increase anxiety and, for some children and adolescents, may lead to a loss of emotional control and intense behavioral reactions often described as “meltdowns” (Elvèn, 2009; Statped, 2022). When the tolerance threshold is reached, this is usually visible in the child's or adolescent's behavior. Acute signs may include self-harm, aggressive behavior, and panic attacks, while long-term consequences may include anxiety disorders, depression, and eating disorders (Elvèn, 2009). Before implementing interventions, it is therefore important to understand what happened beforehand and what contributed to triggering the behavior (Statped, 2022).

Protective and calming factors can help reduce the impact of fundamental stressors. These include predictability and structure; careful consideration of sensory sensitivities; adjusted demands, expectations, and task duration; clear and accessible communication; opportunities to withdraw or take breaks; secure relationships with trusted adults; experiences of mastery; and sufficient time to process without nagging or pressure (Elvèn, 2009; Statped, 2022). These supports are highly relevant for preventing school avoidance, as they help keep the child within a “tolerance window” where participation in school becomes possible and sustainable.

## Inclusion and educational support

Effective inclusion of students with ASD requires structured, predictable, and supportive educational environments that accommodate their unique needs. Inclusion is a multifaceted and dynamic concept with various interpretations, making it a complex phenomenon to define and analyze (Arnesen, 2017). There is no universally accepted standard for what constitutes inclusion or what it means to participate meaningfully in educational and social contexts. Experiences of being included or excluded are deeply personal, with varying emotional and psychological impacts.

For some individuals, inclusion may not always equate to a positive experience if the environment is not genuinely responsive to their needs; conversely, what might appear externally as “separate” provision can be experienced as protective or supportive. The significance of inclusion or exclusion is therefore contingent on personal relevance and individual perception, underscoring the need for educators to engage in dialogue with students and to be attentive to both verbal and non-verbal expressions of their experiences (Arnesen, 2017).

Central to inclusion is the recognition of every child's right to be seen, heard, and valued within the educational community. This is both a legal and ethical imperative that requires educators to exercise professional judgement grounded in conscious decision-making and ethical reflection (Solli, 2017). Schools have a responsibility to cultivate learning environments that promote equity, respect, and participation regardless of students' cognitive abilities, cultural backgrounds, or learning profiles (Håstein and Werner, 2004).

In inclusive classrooms, adapted instruction must address both academic and social dimensions of learning. Students with ASD often have particular needs related to stability, structure, and sensory accommodation, necessitating carefully designed instructional strategies that promote both engagement and wellbeing (Nilsen, 2004). Statped's “7 Hs” framework provides practical guidelines aimed at enhancing predictability and reducing stress for students with ASD (Statped, 2021, 2022). It emphasizes clear expectations, visual supports, and consistent routines to foster structured learning environments.

Structure, predictability, and clarity are critical for facilitating participation in both academic and social activities. Well-defined routines and systems help students understand what will happen, what is expected of them, and how to succeed, thereby reducing stress and promoting feelings of competence. Visual supports—such as object cues, symbols, pictograms, visual schedules, or written instructions—are key tools for making expectations concrete and predictable (Statped, 2021).

The “7 Hs” framework responds to seven guiding questions:

1. What should I do?—Clearly defining the specific task or activity to provide clarity on expectations.
2. Where should I be?—Identifying the physical location where the activity will take place to reduce uncertainty about transitions.
3. Why should I do it?—Explaining the purpose or relevance of the task to enhance motivation and understanding.
4. How should I do it?—Providing explicit instructions or steps to guide task completion effectively.
5. Who should I do it with?—Clarifying whether the activity is to be done individually, in pairs, or as part of a group to support social engagement.
6. How long should I do it?—Establishing clear time frames or session durations to manage expectations and reduce anxiety about task length.
7. What should I do afterward?—Outlining subsequent activities to prepare students for transitions and maintaining a sense of continuity throughout the day.

By systematically addressing these questions, educators can create a learning environment that reduces uncertainty and supports self-regulation, which in turn can contribute to lower stress levels and reduce the risk of school avoidance among students with ASD.

### Teacher–student relationships

Positive teacher–student relationships are fundamental to the academic, social, and emotional development of students with ASD and play a critical role in creating inclusive educational environments where students feel valued, understood, and supported. Emotional and organizational support provided by teachers can significantly enhance students' sense of belonging, which is a key protective factor against school avoidance and disengagement (Havik, 2018).

Teachers serve as emotional anchors within the classroom, providing scaffolding that helps students navigate the complexities of school life. This involves creating an environment characterized by consistency, predictability, and empathy—elements that are particularly vital for students with ASD, who often experience challenges with emotional regulation, social communication, and adapting to change. Consistent and empathetic interactions help establish psychological safety, which is foundational for learning and personal growth.

Developing trust and connection with educators can be particularly challenging for some students with ASD due to difficulties interpreting social cues, understanding relational dynamics, and managing anxiety in social contexts. Teachers attuned to these challenges can play a transformative role by adopting relational approaches that prioritize patience, clarity, and respect. This includes using explicit instructions, visual support, structured routines, and regular, constructive feedback that is sensitive to the student's communication preferences and emotional needs.

Positive teacher–student relationships can buffer the impact of stressors encountered in the school environment. When teachers demonstrate genuine interest, warmth, and a non-judgmental attitude, they help mitigate feelings of isolation, frustration, and anxiety. This supportive dynamic fosters a classroom climate where students feel secure enough to take academic risks, engage with peers, and participate actively. For students with ASD, such relationships may be especially important for preventing school avoidance, as feeling understood and supported by a trusted adult can make attending school more manageable.

Havik (2018) identifies three critical dimensions of support that contribute to strong teacher–student relationships:

- Emotional support—Care, warmth, and empathy, fostering a sense of connection and belonging.
- Organizational support—Clear routines, predictable structure, and a well-organized learning environment.
- Learning support—Differentiated instruction, constructive feedback, and opportunities for mastery that promote competence and intrinsic motivation.

A robust sense of belonging within the school community has been identified as a protective factor against school avoidance and dropout. Students who feel connected to teachers and peers are more likely to attend regularly and engage in both academic and social activities (Havik, 2018). For vulnerable students, including those with ASD, strong social support in school can reduce the impact of academic and social stressors and thereby lower the risk of withdrawal and school avoidance.

Collaboration with families is also integral to teacher–student relationships. Parents and caregivers possess invaluable insights into the child's strengths, challenges, and support needs. Teachers who communicate and collaborate effectively with families are better equipped to provide consistent support across home and school contexts, increasing predictability and stability in the child's everyday life.

## Collaborations

Collaboration between schools, families, and support services is essential for fostering the academic, social, and emotional development of students with ASD and for preventing and reducing school avoidance. Effective collaboration is built on open, transparent communication and a shared understanding of the student's strengths, needs, and aspirations (Drugli and Nordahl, 2016). Such partnerships make it possible to develop tailored interventions that respond to the individual characteristics of each student.

Within the school context, teachers play a pivotal role in establishing and maintaining supportive relationships and in coordinating efforts with other professionals. Developing positive teacher–student relationships, as discussed above, is an ongoing process that requires intentional effort, empathy, and professional reflection (Bergkastet et al., 2011). Collaboration also involves recognizing parents as equal partners whose experiences and perspectives are crucial in understanding the child's behavior across contexts.

A clear definition of collaboration is helpful: collaboration can be understood as a reciprocal, goal-oriented process in which professionals and families share information, responsibilities, and decision-making to support the student's learning and wellbeing. When collaboration functions well, it can minimize school avoidance by ensuring that early signs of stress and avoidance are identified and responded to promptly, supports at home and school are coordinated and consistent, adjustments to the learning environment are meaningful and acceptable to the student, and all stakeholders work toward common goals with clearly defined roles.

Research on professional collaboration in inclusive education underscores the importance of shared planning time, role clarity, and mutual respect among professionals (e.g., Da Fonte and Barton-Arwood; Stone and Charles, 2018; Ní Bhroin and King, 2020; Baum and Schader, 2018). For students with ASD who are at risk of school avoidance, such collaboration is particularly important in designing and implementing gradual re-entry plans, individual education plans (IEPs), and coordinated support strategies.

Where all are part of an inclusive education system, as articulated in Article 24 of the UN Convention on the Rights of Persons with Disabilities, students should be supported to reach their full potential through access to highly qualified teachers and coordinated professional support.

### Interdisciplinary collaboration

Collaboration with external support systems becomes especially critical when school avoidance is severe, longstanding, or complex. When school-based measures alone do not lead to sufficient improvement, a multidisciplinary approach is needed, where different professionals contribute their expertise to develop comprehensive, individualized support plans (Havik, 2018). This support system may include the Educational-Psychological Service (PPT), general practitioners, school health services, child welfare services, and specialized mental health services.

Absenteeism and school avoidance can stem from a wide range of underlying issues, including mental health difficulties (e.g., anxiety, depression, trauma), physical health problems, family dynamics, social challenges, and neurodevelopmental conditions such as ASD. These factors often intersect, making it difficult for schools alone to identify and address the root causes. External services can provide cognitive and psychological assessments, health evaluations, and, when needed, access to specialized therapeutic interventions.

Effective collaboration requires a well-coordinated, interdisciplinary approach. Clear communication channels, shared goals, and regular follow-up meetings are important to ensure that support is coherent and responsive to the student's changing needs. Fragmented or poorly coordinated efforts risk gaps in support and confusion among stakeholders, which, in turn, may undermine trust and the effectiveness of interventions.

When a student has been absent for an extended period, reintegration becomes increasingly challenging as academic gaps, social isolation, and psychological distress deepen. This underscores the importance of early intervention and timely involvement of external services—preferably when concerns about attendance first emerge rather than after patterns of avoidance have become entrenched (Havik, 2018).

A strong and positive school–home partnership is an essential component of this interdisciplinary approach. Parents play a crucial role in understanding the factors contributing to their child's school avoidance and in implementing strategies at home. Collaboration should be characterized by mutual respect, open communication, and a non-judgmental stance, recognizing that school avoidance is usually the result of complex, interacting factors rather than simple lack of motivation.

### School–home collaboration

A strong and effective school–home partnership is crucial for supporting students with ASD in maintaining regular school attendance and addressing school avoidance when it arises. Munkhaugen (2019) emphasizes that such partnerships are central not only for facilitating engagement but also for identifying early signs of emerging difficulties and intervening before patterns of avoidance become entrenched.

Parents and teachers each hold unique and complementary knowledge about the child. Parents have deep insight into the child's developmental history, emotional needs, and everyday functioning at home, while teachers observe how the child copes with academic demands, peer interactions, and the structure of school (Drugli and Onsøien, 2010). Because children with ASD may display different behaviors in different contexts, information from both settings is needed to gain a full understanding of their needs (Ashworth et al., 2025).

Moreover, parents of autistic children with mental health difficulties described feeling constantly overwhelmed, exhausted, and largely left to manage their child's needs without adequate professional support (Balik and Ozgun, 2024). These challenges had wide-ranging effects on the entire family, creating strain in relationships, disrupting daily routines, and contributing to significant stress and isolation. Ashworth et al. (2025) highlight major gaps in available services and underscores the need for earlier, more accessible, and autism-informed mental health support that is responsive to both the child and the broader family context.

Through consistent, open, and constructive communication, schools and families can create a cohesive support system that spans home and school. Positive collaboration fosters mutual trust, respect, and a shared sense of responsibility, making it more likely that interventions will be individualized and effective (Drugli and Nordahl, 2016). Parents of children who display school avoidance often highlight the importance of early dialogue to ensure that necessary adjustments and accommodations are put in place before the child's difficulties escalate (Havik, 2018; Ashworth et al., 2025).

School avoidance can increase in the absence of appropriate accommodation, particularly when students with ASD do not receive individualized support that addresses their sensory, cognitive, emotional, and social needs. At the same time, research indicates that many students themselves report social acceptance and belonging as more important than academic demands when explaining their absenteeism; academic challenges may play a relatively smaller role compared to experiences of exclusion, bullying, or lack of peer relationships (Havik, 2018; Munkhaugen, 2019). Effective collaboration with families must therefore attend to both the social and academic aspects of schooling.

Accommodations may include adjustments to the curriculum, sensory-friendly classrooms, individualized schedules, and targeted social-emotional support. Crucially, students need to feel seen, heard, and genuinely valued by their teachers. For students with ASD, who may be particularly sensitive to feelings of exclusion or misunderstanding, such recognition can be decisive in whether they experience school as a safe place or as a source of stress (Balik and Ozgun, 2024).

Supporting students with ASD, especially those at risk of school avoidance, is a dynamic process that demands continuous collaboration, flexibility, and responsiveness from both schools and families. Regular meetings, progress monitoring, and open lines of communication are essential. Interventions should be designed with the student's best interests at the center, focusing on strategies that improve the situation rather than unintentionally increasing stress or anxiety (Havik, 2018).

## Theoretical implications for practice

Theoretical insights into ASD and school avoidance highlight the importance of individualized, student-centered approaches that consider sensory sensitivities, communication differences, executive functioning challenges, and the need for structure and predictability (Siller et al., 2024). Students with ASD may experience heightened responses to environmental stimuli, difficulties in navigating complex social interactions, and challenges in coping with unexpected changes. Schools therefore need flexible and personalized strategies that support both learning and emotional regulation (Balik and Ozgun, 2024).

Early identification and timely intervention are central. Systematic screening and monitoring of attendance patterns, emotional wellbeing, and classroom participation can help identify students at risk. Theoretical frameworks related to inclusive education, neurodiversity, and behavioral psychology provide guidance for designing proactive interventions that seek to adjust environments rather than merely changing the child (Siller et al., 2024).

Ongoing professional development is essential to maintain and develop teachers' competence in supporting students with ASD. Training should focus on differentiated instruction, positive behavioral support, sensory-informed pedagogy, and effective communication with students and families. Professional learning communities within schools can facilitate shared reflection, exchange of experience, and coherence in practices across classrooms and year groups (Vivanti et al., 2025).

Integrating theoretical knowledge with practical application is fundamental for creating educational environments where students with ASD can succeed academically, socially, and emotionally. This integration also means viewing school avoidance not solely as a behavioral problem but as a multifaceted phenomenon influenced by biological, psychological, and environmental factors. Adopting such a holistic perspective encourages compassionate, individualized approaches that seek to address root causes—such as anxiety, sensory overload, or lack of belonging—rather than focusing exclusively on attendance as an outcome (Vivanti et al., 2025).

Concrete ways in which bridging theory and practice can help minimize school avoidance include:

- designing structured, predictable classroom routines informed by knowledge of executive functioning and sensory processing;
- using evidence-based approaches (e.g., CBT-informed strategies, visual supports, graded exposure) to reduce anxiety around school;
- developing individualized plans in collaboration with students, families, and external services;
- and systematically evaluating and adjusting interventions based on ongoing observation and feedback.

In this process, teachers, special educators, school leaders, and external professionals all have important roles, while students and families should be active partners in identifying priorities and evaluating what works.

## Competence

Schools should function as dynamic environments where staff engage in continuous professional development, both individually and collaboratively. Such development is essential for fostering inclusive practices and for ensuring that educators are equipped to meet the diverse needs of all students, including those with ASD and other neurodevelopmental differences (Balik and Ozgun, 2024).

Meld. St. 6 (2019)–(2020) highlights the importance of accessible special education expertise within schools. Children who require special accommodation benefit from direct access to specialized competence. Importantly, this expertise benefits not only students with special educational needs but also the broader student population, by promoting differentiated instruction and a deeper understanding of diverse learning profiles.

The White Paper emphasizes increasing competence in pedagogical practices to provide high-quality education tailored to individual needs, as well as the importance of interdisciplinary collaboration. Collaboration between teachers, special educators, school psychologists, speech and language therapists, and other support personnel is necessary to ensure holistic support that promotes both academic and socio-emotional development.

Meld. St. 6 (2019)–(2020) also states the government's intention to ensure that educational expertise is available where children and students are—meaning that specialized support should be embedded in everyday learning environments rather than isolated in separate arrangements. This aligns with the principles of inclusive education and universal design for learning, where flexible, accessible learning environments are designed from the outset.

In line with this, the Norwegian Parliament's Decision No. 901 of June 13, 2017, mandates that students with special educational needs are entitled to instruction provided by professionals with recognized and relevant qualifications. This underscores that students with additional needs should have access to high-quality education delivered by educators with formal training in pedagogy and special education.

A concern raised in Meld. St. 6 (2019)–(2020) is the extensive use of teaching assistants without formal pedagogical or special education qualifications in special education. While teaching assistants can play a valuable supportive role, relying heavily on unqualified staff can undermine the quality and effectiveness of interventions. The White Paper argues for stricter regulation and for investment in the recruitment, training, and retention of qualified special education professionals.

Ensuring high-quality education for students with special educational needs therefore requires an integrated approach that combines professional development, interdisciplinary collaboration, and equitable access to specialized expertise. By prioritizing competence development and embedding specialized support within mainstream educational settings, schools can create learning environments where all students are supported to reach their full potential, both academically and personally (Balik and Ozgun, 2024).

## Conclusion

The intersection of autism spectrum disorder and school avoidance presents a complex challenge that requires a multifaceted theoretical and practical response. This article has highlighted the diversity of ASD presentations and the range of factors that can contribute to school avoidance, including social communication differences, sensory sensitivities, executive functioning challenges, and co-occurring mental health conditions.

Addressing school avoidance among students with ASD demands strategies that prioritize emotional wellbeing, sensory accommodations, and structured, predictable environments. Distinguishing school avoidance from truancy is crucial, as it underscores the need for supportive rather than punitive responses and for interventions that are sensitive to underlying anxiety and stress.

Bridging theoretical insights with practical applications involves translating knowledge about ASD, stress, and learning into concrete educational practices. This includes implementing visual and structural supports, fostering strong teacher–student relationships, engaging in meaningful school–home collaboration, and coordinating with external services when needed. These practices can help reduce stress, strengthen students' sense of belonging, and make school attendance more manageable and meaningful for students with ASD.

Teachers, special educators, school leaders, and external professionals all contribute to creating environments in which students with ASD can thrive. When theory and practice are integrated in a thoughtful and systematic way, educational settings are more likely to promote engagement, reduce school avoidance, and support the overall development and long-term wellbeing of students with autism.

## Data Availability

The original contributions presented in the study are included in the article/supplementary material, further inquiries can be directed to the corresponding author.
